# Development of a patient-specific epicardial guide for ventricular tachycardia ablation surgery using high-consistency rubber silicone molding

**DOI:** 10.1186/s12938-025-01435-z

**Published:** 2025-11-10

**Authors:** Rani Kronenberger, Mara Candelari, Ida Anna Cappello, Leire Viana Uribe, Giacomo Talevi, Luigi Pannone, Andrea Maria Paparella, Bastien Chabriere-Navarro, Cinzia Monaco, Ivan Eltsov, Robbert Ramak, Gian Battista Chierchia, Mark La Meir, Ali Gharaviri, Carlo de Asmundis

**Affiliations:** 1 Cardiac Surgery Department, Vrije Universiteit Brussel, Universitair Ziekenhuis Brussel, Laarbeeklaan 101, Brussels, Belgium; 2 Heart Rhythm Management Centre, European Reference Networks Guard-Heart, Heart Rhythm Research Brussels, Postgraduate Program in Cardiac Electrophysiology and Pacing, Universitair Ziekenhuis Brussel, Vrije Universiteit Brussel, Brussels, Belgium; 3 Polymer Systems Technology, Lincoln Rd, High Wycombe, HP12 3RF UK

**Keywords:** 3D printing, 3D-printed molds, High-consistency rubber silicone, Ischemic scar, Patient-tailored surgical guide, Radiofrequency ablation, Rubber molding, Ventricular tachycardia

## Abstract

**Background:**

Concomitant epicardial ablation of ventricular tachycardia (VT) remains a clinical challenge in cardiac surgery due to the need for intra-operative mapping. A preoperative patient-tailored epicardial guide could provide an intraoperative ‘blueprint’ of arrhythmogenic target substrate, thereby facilitating the workflow. Thus far, no 3D printing material or technique has fully met the requirements for this application. This study explores the feasibility of high-consistency rubber (HCR) silicone molding to produce low-cost, customized guides for VT scar ablation.

**Methods:**

An inverted mold was created in Meshmixer using merged LGE–CMR and cardiac CT images of a 61-year-old VT patient, and printed using fused deposition modeling. HCR silicone was milled, sculpted, and trimmed to fit the negative mold. The guide was cured, subjected to autoclaving, and bench-tested on an ex vivo porcine heart model using radiofrequency and cryo-ablation. Various durometers and thicknesses were tested to determine the optimal fit for our application.

**Results:**

Five surgical guides were made using NuSil^™^ MED-4072 and MED-4080 silicone (thickness range: 2.0–3.4 mm). Models with 2.1–3.0 mm thickness and 70 Shore A hardness achieved the best balance between flexibility and rigidity for application on a beating heart. The thinnest model (2.0 mm) was too pliable for stable placement. The guides withstood autoclaving and ablation procedures (radiofrequency; cryo-energy) without deformation or compromising structural integrity.

**Conclusions:**

The HCR silicone molding technique allows for the production of flexible, cost-effective epicardial guides for VT ablation, minimizing the need for a full electrophysiology team throughout the entire procedure.

**Supplementary Information:**

The online version contains supplementary material available at 10.1186/s12938-025-01435-z.

## Introduction

Post-infarction ventricular tachycardia (VT) carries a high risk of morbidity and mortality due to malignant re-entrant arrhythmias arising from post-infarction ischemic scars [1–4]. In drug-refractory patients with failed catheter ablation—approximately 20–50% of cases—epicardial surgical cryo-ablation emerges as a promising therapeutic approach [2]. Surgical VT ablation requires real-time intraoperative electro-anatomic mapping to delineate the regions of interest by the electrophysiology (EP) team. However, significant procedural challenges persist.

First, epicardial VT ablation in patients undergoing concomitant cardiac surgery remains a clinical challenge due to the lack of intraoperative guidance, as mapping cannot be performed on the arrested heart. During concomitant ablation on the beating heart, air in the open pericardium creates impedance that reduces signal quality. As a result, concomitant epicardial ablation is rarely performed, despite clinical indication and surgical access.

Second, the organizational challenge of coordinating simultaneous availability of both EP and surgical teams for a stand-alone surgical ablation procedure persists. As described by Calkins et al. in 2012 as a “logistical nightmare”, “both experts should be available in the same hospital, on the same day, at the same time, and for as long as 8 h (the longest procedure in this report required 505 min)” [5]. This process significantly lengthens anesthesia- and operating time*.* More than a decade later, no effective solution has emerged to overcome this logistical barrier. In addition, the efficacy of epicardial mapping could be compromised by several technical and physiological factors. Conventional epicardial mapping tools, originally engineered for endocardial applications, exhibit reduced precision when applied epicardially [6]. Moreover, the absence of fixed visual references and the biomechanical challenges posed by the continuously contracting myocardium further reduces accurate scar tissue localization. Moreover, signal distortion caused by metallic surgical instruments that interfere with magnetic field-based imaging modalities, and variability in tissue impedance which adversely affects electrogram quality [6]. Finally, spatial discrepancies between electro-anatomic mapping and pre-operative imaging of scar tissue further limit the reliability of voltage maps in guiding ablation. These limitations are key drivers of incomplete ablation and repeat interventions in a particularly vulnerable patient population. This creates a (preventable) clinical and financial burden on patients and healthcare systems.

Thus, there is a need for a reliable method to improve intraoperative identification and targeting of arrhythmogenic tissue during cardiac ablation, potentially reducing reliance on a full EP team and real-time mapping. To address these limitations, we previously developed a novel, patient-specific surgical guide that converts pre-operative data into a tactile, intraoperative tool [7]. The guide envelopes the epicardial surface and serves a dual function: (I) as a navigational aid by delineating scar and arrhythmogenic regions to improve targeting accuracy for ablation on the arrested heart or during stand-alone surgical ablation; and (II) as a protective barrier for non-target tissues such as the coronary arteries.

Material selection is a critical consideration in the development of the guide. Criteria include biocompatibility, structural resilience, and capability of withstanding the mechanical and thermal stresses associated with sterilization and ablation procedures. Common materials for surgical three-dimensional (3D) printing include resins, such as PolyJet and stereolithography–liquid crystal display printed photopolymers. However, these materials are UV-cured, making them brittle, with potential cytotoxicity from uncured monomers or leaching of potentially hazardous compounds [8]. In addition, the associated additive manufacturing processes are often complex, labor-intensive with a risk of printing failures and the need for well-designed support structures.

This study proposes the use of high-consistency rubber (HCR) silicone, a technique and material with a well-established safety profile and long-term biocompatibility in clinical applications [9]. When combined with fused deposition modeling (FDM) mold printing, HCR silicone offers superior elasticity, tensile strength, and thermal stability over a broad temperature range, making it well-suited for both cryo-ablation, radiofrequency ablation, and pulsed field ablation modalities.

## Results

### Phantom manufacture

A Polylactic Acid (PLA) mold was printed using FDM technology, with 200 μm layer thickness resolution mode. The region of interest (surgical delineation) matched the target dimensions from the design software. The printed model weighed 300 g.

### Device fabrication check

Five surgical guides were fabricated using NuSil™ MED-4072 and MED-4080 silicone (thickness range: 2.0–3.4 mm). Specifications per model are shown in Table 1. The total process time from initial imaging to the final cured surgical guide was approximately 2 days for the first proof-of-concept, with an estimated cost of €80 per guide, and a total silicone material usage of approximately 1000 g. The surgical guide fits accurately and securely on the phantom model.

**Table 1:** Specifications of the models created in this study

Guide	Material	Shore A Hardness	Thickness (mm)
Model 1	MED 4072	70 Shore	2.0
Model 2	MED 4072	70 Shore	2.1
Model 3	MED 4072	70 Shore	3.0
Model 4	MED 4072	70 Shore	3.4
Model 5	MED 4080	80 Shore	3.0

### Bench testing

#### Material integrity assessment

Molds produced through this method showed a consistent appearance. A macroscopic inspection of all disinfected and autoclaved models revealed no defects. None of the models exhibited cracking, breakage, or color changes.

### Fit for application and folding mechanism

Models with a thickness of 2.0–3.0 mm and 70 Shore A hardness provided a favorable balance between flexibility and rigidity, making them well-suited for application on a beating heart. Models thinner than 2.0 mm would be overly pliable, resulting in inadequate stability and a tendency to lose consistent contact with the epicardial surface. Model 5 with Shore A 80 hardness was too rigid, as this hardness in combination with a 3.0 mm thickness would fail to conform to the heart’s motion and remain largely static during the cardiac cycle.

Excess material at the apex contributed to bulkiness, which prevented full insertion in a trocar, especially relevant with the thinnest models (model 1, model 2). While other models with increased thickness and hardness offered better stability, they were either too thick or rigid due to excess apical base material, making them difficult to insert through the trocar and unsuitable for use on a beating heart.

### Bench testing on animal hearts

**Fig. 1:**
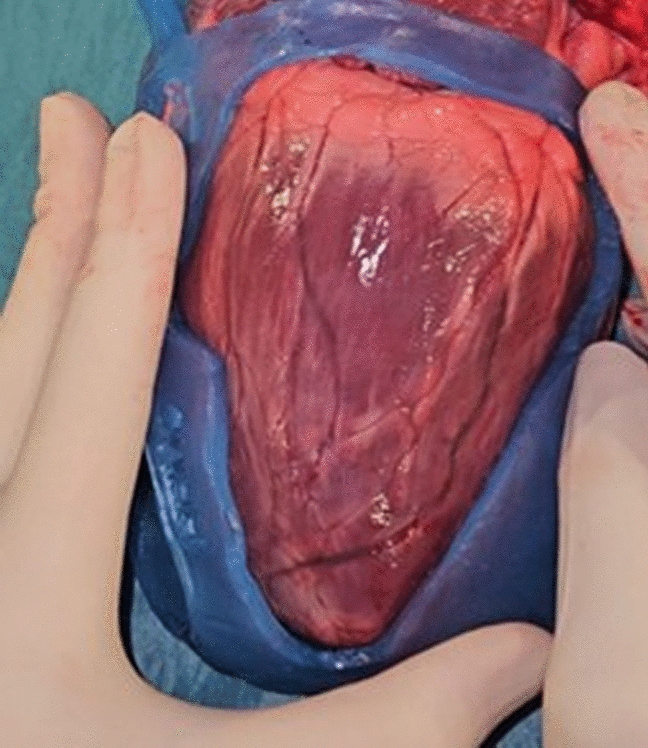
Surgical guide on ex vivo porcine heart showing VT scar region

**Fig. 2:**
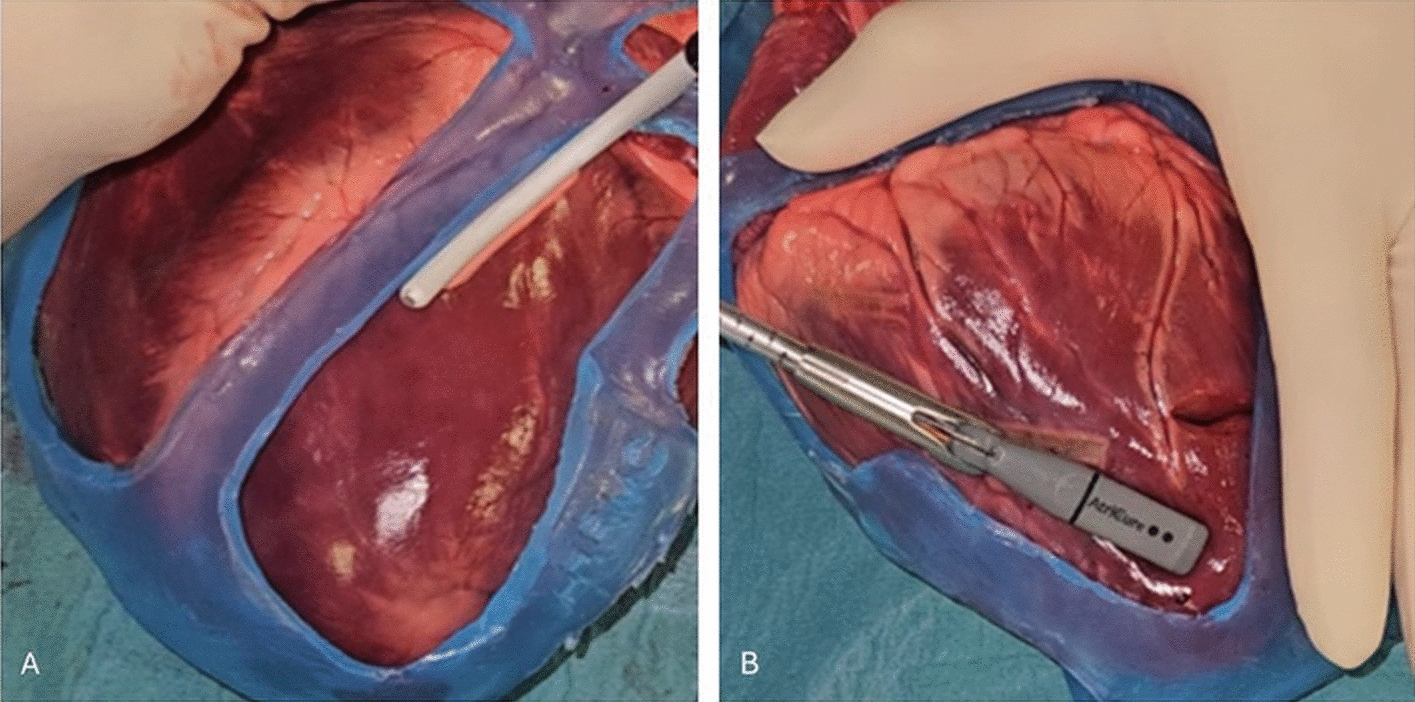
**A** Cryo-ablation lesions on ex vivo porcine heart. **B** Radiofrequency ablation on ex vivo porcine heart

Ablation procedures were performed on four ex vivo porcine hearts (Figs. 1, 2). Two energy sources were utilized for all ablation procedures (radiofrequency, cryo), and standardizing the exposure time and temperature ensured consistency across experiments, closely simulating clinical conditions. The silicone showed no signs of breakage or color change after radiofrequency or cryo-ablation near the guide border, or on non-target tissue below the guide. No deformations were seen after thermal stress.

## Discussion

This preliminary study is the first to assess the use of HCR wrapping for patient-specific guides in VT scar ablation.

Innovative solutions are needed to precisely identify and target complex epicardial substrate during concomitant open-chest surgery—enabling accurate localization on the arrested heart—and in stand-alone procedures, where prolonged surgery and the need for an EP team in the room present additional challenges. Epicardial guides would allow the surgeon to improve navigation, accuracy, safety, and therapeutic efficacy in difficult-to-treat ablation patients.

Herein, we aimed to develop a feasible, cost-effective, and rapid method for creating epicardial surgical guides, focusing on early feasibility and technical viability. Compared to traditional 3D printing methods, HCR molding offers an efficient and affordable alternative that yields promising potential.

### Rationale behind silicone and molding

The emergence of 3D printing technologies in the last decade has created abundant application opportunities in the field of precision medicine and surgical education [10]. However, 3D printing resins face challenges, such as brittleness, poor dimensional accuracy and low mechanical strength, especially after post-processing and sterilization, as well as limited material options, high costs, and lack of standardization [11–13]. In addition, uncured monomers or leachates may release cytotoxic byproducts, further complicating their use in surgical environments [14]. To overcome the limitations of traditional 3D printing resins, we chose a silicone-based solution—an elastomer widely used in the medical field [15]. Some silicones are approved for long-term implantation and have a proven track record in dynamic (cardiovascular) surgical environments. With its flexibility, durability, stability, versatility, and resistance to extreme temperatures, silicone can be fully customized to meet the specific needs of this epicardial application.

Although silicone modeling has been used to create customized molds [16–18], no studies have fabricated silicone specifically as an epicardial guide, posing unique challenges in identifying a material that meets all necessary criteria. Material choice is a critical factor to the success of a patient-specific medical device. Properties such as silicone hardness and flexibility help the guide maintain shape and function despite the heart’s constant motion, which prevents gaps that could lead to uneven lesions.

We also considered liquid silicone rubber molding, a widely used technique in medical device manufacturing and studies. However, this method requires creating an inverted mold with several components (multi-part mold system, vent channels, and silicone channels) that must be precisely aligned and securely connected using interlocks to prevent movement. This makes the process significantly more labor-intensive and less suitable for a streamlined, efficient, and rapid customized workflow for ablation treatments. Another barrier is that the liquid silicone for our required durometer would be too viscous at room temperature, making injection into the mold difficult and prone to air entrapment. We also considered direct 3D silicone printing, a more novel approach. However, it was not our preferred choice due to potential challenges with support structures, layer adhesion, and viscosity control, which could compromise integrity and make the process tedious.

An advantage of silicone wrapping is that sculpting results in a smoother inside surface due to the natural smoothness of milled silicone; unlike 3D printing, which can create rougher surfaces. This technique also allows customization using different silicones, curing times, and pigments to meet tailored requirements.

### Thermal insulation and procedural safety

The guide manufacturer is responsible for ensuring compliance with design requirements (form, fit, and function) throughout the entire device’s life cycle [19]. A critical consideration in the development of surgical guides is their thermal insulation performance under ablative stress. These properties are vital for two primary reasons: ensuring procedural safety during targeted ablation and protecting adjacent, non-target anatomy from thermal injury. The material’s electrical non-conductivity reduces the risk of unintended heating when ablation instruments come into accidental contact with the guide’s edges—a scenario that can result in localized temperatures as high as 70–90 °C during radiofrequency ablation or below 0 °C during cryo-ablation, potentially causing collateral tissue damage. Due to the lack of conductivity data in the material datasheet, preliminary tests were conducted simulating accidental instrument-guide contact. These experiments confirmed that the guide effectively prevented thermal injury to underlying tissue. However, further comprehensive thermal and mechanical characterization—particularly post-processing and sterilization—remains necessary to validate the material’s suitability for clinical application.

### Design considerations

Guide stability on the epicardial surface is of critical importance to ensure precise lesion placement. Material testing indicated that silicone with a 70 Shore A hardness and 2–3 mm thickness provided an optimal balance between flexibility and intraoperative stability on the beating heart. In addition, preliminary foldability tests were conducted to assess suitability for minimally invasive approaches, revealing that the current guide designs are too bulky for efficient trocar introduction, largely due to excess material at the apex base. However, this limitation is expected to be less significant in smaller, target-specific guide designs. Future work should explore alternative silicones and ensure uniform thickness without excess material. Moreover, to facilitate insertion and removal of the guide through a trocar, incorporating a secure locking system with small tabs on the lateral sides that click when folded might prove useful.

Finally, an important challenge in epicardial guide use for minimally invasive stand-alone ablation is achieving stable anchoring of the model on a beating heart. Potential solutions we considered include micro-suction ports, adhesive surfaces, or specialized gripping materials.

### Broader clinical applicability and translational pathway

While the guide in this feasibility study was designed to cover a broad scar region, the end objective is to use preprocedural input from the EP to incorporate predefined ablation targets in form of ‘windows’ in the mask, directly providing the surgeon with an ablation roadmap or lesion set. By providing intra-operative guidance, it could enhance precision and reduce reliance on full real-time mapping and a complete EP team, since lesions are identified pre-operatively. However, the customization of these ablation openings requires further finetuning as they depend on patient pathology, surgical approach (open-chest or minimally invasive), and the type and size of the catheter used.

A crucial early step in clinical translation is assessing biocompatibility and characterizing the sterilized silicone material to confirm its suitability, thermal stability, mechanical integrity, and overall safety for clinical use. Preclinical validation is essential to rule out risks, such as loose fragments or allergic reactions, and to assess the guide’s fit, stability, efficacy and precise electro-anatomic alignment. Swine models are commonly used due to physiological similarity to humans and comparable heart rates, beginning with open-chest studies and advancing to minimally invasive approaches [20]. For these experiments, subject-specific guides are fabricated from pre-operative cardiac CT. While device alignment partially relies on visible anatomical landmarks (aorta, pulmonary artery ring, and left ventricular apex)—which are accessible via both open-chest and thoracoscopic approaches—epicardial electro-anatomic mapping is still needed during early translational phases. Alignment accuracy can be quantified by measuring the percentage overlap between pre-operative targets (scar and mapping) and post-ablation areas confirmed by mapping, and post-explant TTC staining and histology.

The expected benefits—allowing concomitant VT ablation, improved consistency, shorter procedures, fewer repeat ablations—translate into both clinical and economic value. Though regulatory and compliance efforts for clinical translation are significant, they are minor compared to the costs of repeat ablations, prolonged procedures, and hospital stay/visits—especially once integrated into routine practice. While the training and integration of the device into the surgical workflow may initially add time to the procedure, it is expected to reduce overall operative time over the long term—particularly if intraoperative mapping can be reduced, potentially eliminating the need for an EP team in the operating room.

This silicone-based approach can serve as a basis for the engineering and development of other customizable guides. The potential for further applications is vast, as this can be adapted to meet specific mapping and procedural requirements. For example, our group previously developed an epicardial guide for Brugada syndrome ablation, highlighting the arrhythmogenic substrate in the right ventricular outflow tract, based on electro-anatomical mapping data [21]. In addition, we have created guides for coronary artery mapping and optimal bypass target placement during coronary artery bypass grafting [22], and sinus node protection for inappropriate sinus tachycardia ablation. Furthermore, this approach could extend to other (non-)cardiac procedures for anatomical modeling, pre-operative planning, and surgical training.

### Limitations and future considerations

Several limitations should be considered. As this was an early proof-of-concept study, the 3D-printed mold was fabricated from non-biocompatible PLA. Thus, biocompatibility testing, including in vitro cytotoxicity assessments as per ISO 10993–5:2009 (https://www.iso.org/standard/36406.html), as well as post-sterilization material characterization assessments are warranted for future research. Following, in vivo porcine experiments are needed on the beating heart to assess stability, fit, ablation efficacy, and electro-anatomic alignment more in-depth.

## Conclusions

In conclusion, we proposed a cost-effective and rapid method for creating epicardial surgical guides. This preliminary study demonstrated the feasibility and efficacy of high-consistency rubber silicone in producing patient-specific guides for VT scar ablation. This approach could minimize the need for intraoperative mapping, shorten anesthesia- and single-lung ventilation times, and decrease reliance on a full hybrid team. While further optimization is needed, HCR molding shows promise for epicardial guides.

## Materials and methods

**Fig. 3:**
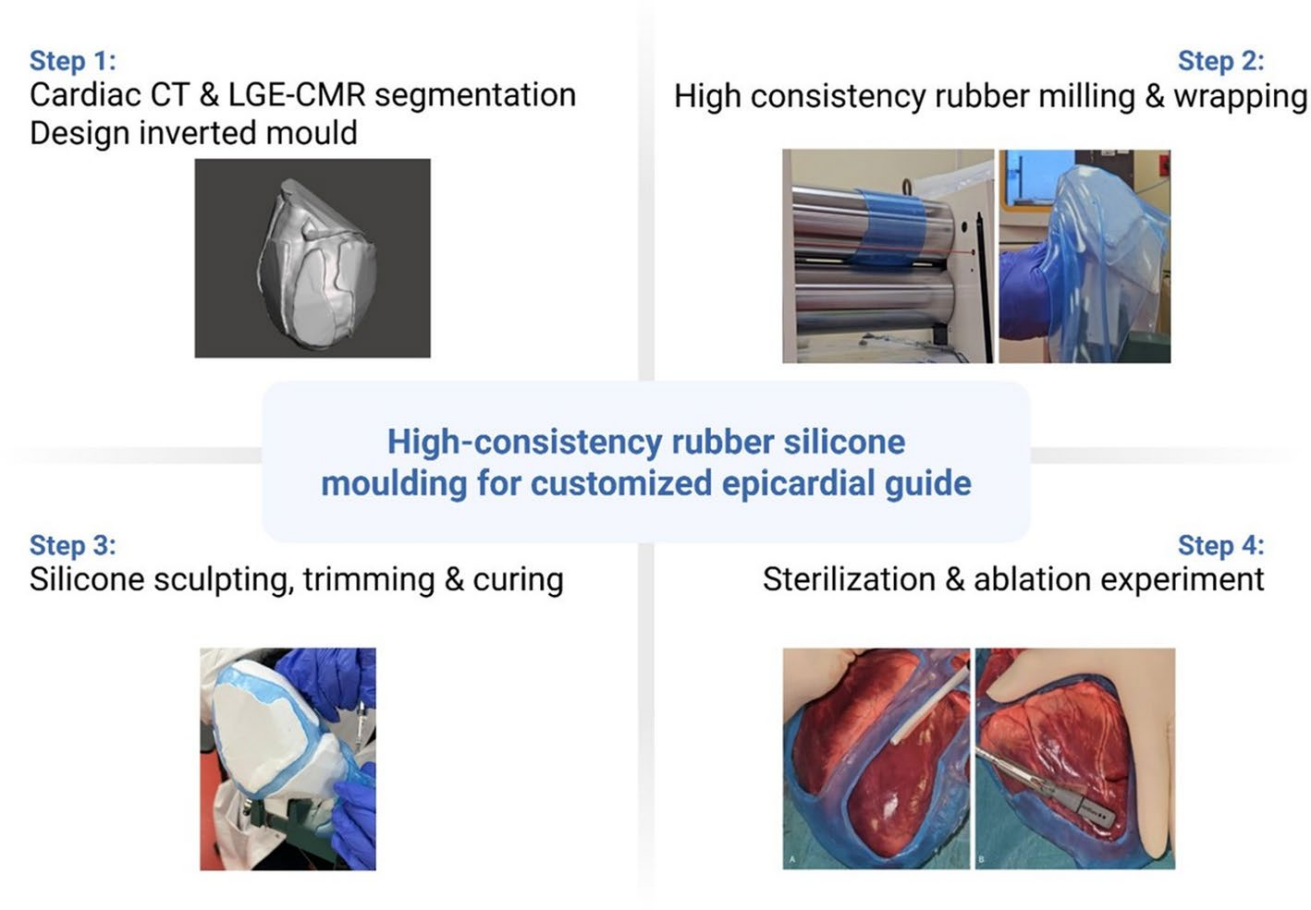
Stepwise approach to this study’s methodology

An overview of the methodology is shown in Fig. 3.

### Patient selection

**Fig. 4:**
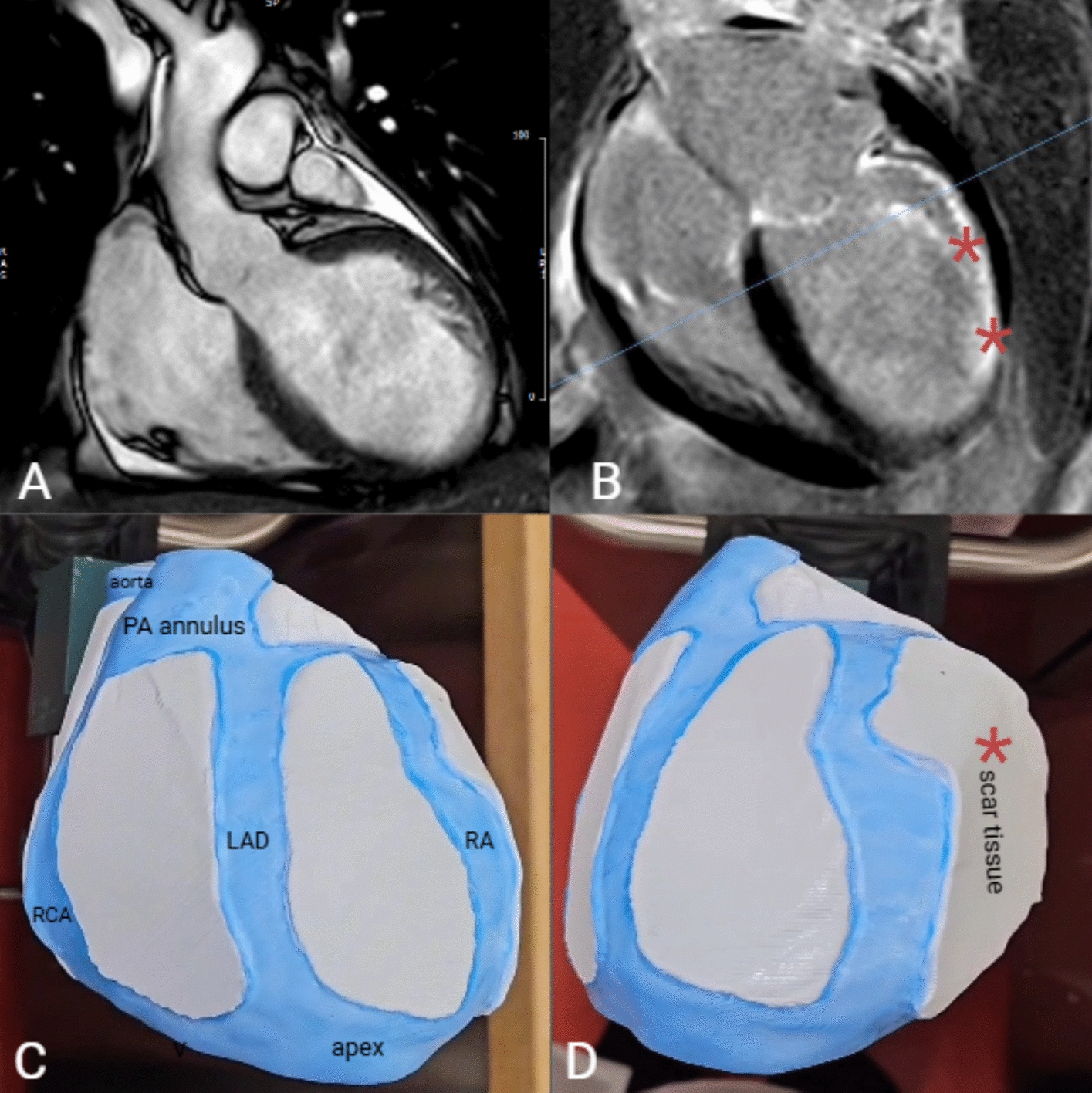
Late gadolinium enhancement cardiac MRI (**A**), with post-infarction scar indicated by red asterisks in (**B**). (**C**, **D)** 3D surgical guide showing anatomical landmarks: aorta, pulmonary artery (PA) annulus, and apex. Coronary protection designed for the right coronary artery (RCA), left anterior descending artery (LAD), and ramus angularis (RA)

Our research builds upon a case previously described by our group [7]. In brief, a 61-year-old man with cardiovascular risk factors and a prior lateral myocardial infarction presented with severe left ventricular dysfunction and drug-refractory recurrent monomorphic VT requiring ablation therapy. Late gadolinium-enhanced cardiac magnetic resonance (LGE–CMR) revealed transmural late gadolinium enhancement, papillary muscle infarction, and akinesia (Fig. 4B).

### Guide specifications

#### Materials

Fused deposition modeling (FDM) printing, an extrusion-based 3D printing technique, was used to print a mold in polylactic acid. This method was selected due to its low cost and high accessibility for rapid prototyping [23].

For the silicone models, we chose ultra-high-performance silicone elastomers with excellent hyperelasticity, tensile strength, and a wide thermal stability range, making them well-suited for both cryo and radiofrequency ablation. Its mechanical properties can be fine-tuned for specific surgical applications. Although widely used in industrial settings, its potential in custom surgical guides remains largely untapped.

We used biocompatible NuSil™ MED-4072 and MED-4080 (NuSil Technology, CA, US). MED-4072 is a durable, high-strength silicone with flexible curing options, fast processing, and safe, non-volatile properties—ideal for surgical guide fabrication. MED-4072 consists of a three-part system. MED-4080 is a two-part, high tear strength silicone elastomer. Table 2 summarizes their main properties.

**Table 2:** Main characteristics and detailed properties of MED-4072 and MED-4080

Typical Properties	MED-4072	MED-4080
Specific Gravity (ASTM D792)	1.22	1.20
Durometer, Type A (ASTM D2240)	70	80
Tensile Strength (ASTM D412)	1,100 psi (7.6 MPa)	1,150 psi (7.9 MPa)
Elongation (ASTM D412)	875%	735%
Tear Strength (ASTM D624)	240 ppi (42.3 kN/m)	225 ppi (39.7 kN/m)
Stress at 200% Strain (ASTM D412)	450 psi (3.1 MPa)	450 psi (3.1 MPa)
Tissue Culture (Cytotoxicity Testing) (USP < 87 > , ISO 10993–5)	Pass	Pass
Elemental Analysis of Trace Metals (ASTM E305)	Pass	Pass

#### Thickness

The primary consideration regarding thickness is that the guide must conform to the anatomy of a beating heart, providing dynamic stability without being excessively pliable. Second, hybrid ablation uses minimally invasive ports, with the widest trocar being 12 mm. To ensure the guide can be introduced via thoracoscopic forceps, it should be flexible enough to pass through a 12 mm trocar. To reflect both these considerations, we initially selected thicknesses ranging from 2.0 to 3.4 mm.

### Surgical guide design

#### Modeling of surgical guide

Our model builds upon the previously published method by our group [7]. This model was created using data from cardiac computed tomography (CT) images for cardiac anatomy and LGE–CMR to pinpoint ischemic scar regions. The scar border was drawn (Fig. 4D), and anatomical landmarks such as the aorta, pulmonary artery annulus, and heart apex were manually connected to the target area (Fig. 4C). Additional reference points, including the right coronary artery, left anterior descending artery, angular artery, and circumflex artery, were incorporated to highlight and protect the coronary arteries from thermal injury (Fig. 4C). After data acquisition and image segmentation, the data sets were processed and merged using 3D Slicer and Mimics, creating a stable surgical guide.

#### Design of the mold

The final patient-tailored guide was then designed. We reconstructed the VT mask’s inner surface using Meshmixer (Meshmicker, CA, USA). The original guide was inverted into a negative mold, preserving the interior bulk. This modified model was converted into an*.stl* file and prepared for 3D printing.

### Surgical guide manufacturing

The guide manufacturing process is detailed in Video 1 (see supplemental material).

#### Mold fabrication

The inverted model was 3D-printed using FDM printing technology (Funmat HT, Intamsys, Shanghai, China). After 3D printing, the 3D mold was evenly sprayed with a release agent (MediMould) and left to air dry for 10 min.

#### High-consistency rubber extrusion

##### Milling

MED-4072 silicone (270 g) was softened through roll milling using an MW15 two-mill roll (Dijatec, Utrecht, Netherlands). The elastomer was re-softened before use to ensure maximum uniformity, as proposed by the material sheet (NuSil, CA, USA).

##### Pigmentation and additives

A blue translucent pigment (MED 40–4900-7; NuSil, CA, USA) was added and mixed by milling. Following, 0.5% inhibitor and 1% catalyst were incorporated. The material was then milled 6–10 times at the preferred thickness to ensure uniform dispersion.

#### High-consistency rubber wrapping

After confirming homogeneity and absence of entrapped air, the milled silicone sheet is removed and kept vertical. It is then carefully placed over the mold, ensuring full coverage without folds. The HCR is then carefully wrapped around the printed model to form the final structure.

#### Sculpting and trimming of excess silicone

The material is sculpted around the guide model, followed by precise cutting with a knife to achieve a smooth surface. Excess silicone was meticulously trimmed away using a scalpel or fine scissors to ensure a precise fit and to maintain consistent thickness throughout the model.

#### Curing

After wrapping, the model (mold and guide) was cured in an oven at 60 °C for 1 h, selected to avoid deforming the mold, achieving 90% cure (T90). To reach full cure (T100), an additional 10 min at 100 °C was applied post-demolding. This ensured the rubber reached the desired hardness and durability.

#### Post-processing

The model was thoroughly inspected for imperfections and checked for quality to confirm it met the necessary specifications for the project. Any excess material or rough edges left from the HCR wrapping were carefully trimmed away using a fine scalpel, ensuring the surface that would come in contact with the epicardium was smooth and even to the touch.

### Disinfection and sterilization

Since the material data sheets did not specify sterilization protocols, we chose pre-sterilization mechanical washing followed by steam autoclaving, the most widely used and cost-effective method in healthcare, and readily available in our hospital. The specimens were cleaned using a 1.5-h washing cycle, which included a pre-rinse, washing with Mediclean Forte and Septoclean, thermal disinfection at ~ 95 °C, and a final drying phase. The samples were steam-sterilized at 134 °C for 4 min using an autoclave with fractionated pre-vacuum in a 60-min cycle at 3045–3101 mbar. After sterilization, they were cooled and left to degas for 12 h. All processes were performed at the University Hospital of Brussels' Central Sterilization Unit.

After disinfection and sterilization, a visual inspection was performed by RK to check for macroscopic defects, such as crazing, cracking, breakage, loose fragments, or color changes.

### Animal heart testing

*Bench testing* was conducted on ex vivo porcine hearts, matched closely to the patient’s heart the guide was built upon, to evaluate the surgical guide’s material fit, and integrity in mimicked ablation conditions. Ex vivo porcine hearts were chosen due to their close anatomical and physiological similarity to human hearts, including comparable size and coronary structure. The silicone guide was manually folded, secured with a surgical tie, and inserted through a 12 mm trocar using thoracoscopic forceps to simulate minimally invasive surgery. The guide was then tested under thermal stress for fit, stability, and deformation resistance. Ablation procedures were simulated with energy delivery in the buffer zone and partially on the guide to mimic accidental thermal stresses (localized heating up to 90 °C), such as those from unintended contact with ablation instruments.

Four porcine hearts were prepared in a surgical field, and each silicone guide was applied to the epicardial surface of the heart. Six ablation lesions were created adjacent to the guide’s border using unipolar radiofrequency ablation and cryo-ablation at separate locations approximately 0.5 cm from the edge. Ablations were performed according to standard clinical settings.: unipolar radiofrequency energy was delivered using the Coolrail® linear pen (AtriCure, Ohio, US) (30W, 40 s, 90 °C), and cryo-ablation was performed using the cryoICE ablation system (AtriCure, Ohio, US) (-72 °C, 120 s energy delivery, 60 s wean). Visual inspection assessed for post-ablation material deformities, breakage, cracking, or color changes after thermal stress.

## Supplementary Information

Additional file 1. Video 1. Workflow for surgical guide creation using FDM printing with high-consistency rubber wrapping

## Data Availability

No data sets were generated or analyzed during the current study.

## Abbreviations

3D: Three-dimensional
FDM: Fused deposition modeling
HCR: High-consistency rubber
LGE–CMR: Late gadolinium-enhanced cardiac magnetic resonance
PLA: Polylactic acid
VT: Ventricular tachycardia
